# Comparing the cost of essential nutrients from different food sources in the American diet using NHANES 2011–2014

**DOI:** 10.1186/s12937-019-0496-5

**Published:** 2019-11-09

**Authors:** Julie M. Hess, Christopher J. Cifelli, Sanjiv Agarwal, Victor L. Fulgoni

**Affiliations:** 1 National Dairy Council, 10255 West Higgins Road, Suite 900, Rosemont, IL 60018-5616 USA; 2 NutriScience LLC, East Norriton, PA 19403 USA; 3 Nutrition Impact, LLC, Battle Creek, MI 49014 USA

**Keywords:** Affordability, Sustainability, Calcium, Vitamin D, Potassium, Dairy, Milk

## Abstract

**Background:**

One reason that some Americans do not meet nutrient needs from healthy eating patterns is cost. Food cost affects how people eat, and healthy diets tend to be more expensive. Cost is also important for diet sustainability. Sustainable eating patterns must be both nutritionally adequate and affordable. The objective of this study was to compare the cost of obtaining shortfall nutrients from different food groups to help identify cost-effective ways Americans can move towards healthy and sustainable eating patterns.

**Methods:**

This analysis used dietary intake data from the National Health and Nutrition Examination Survey from 2011 to 2012 and 2013–2014 (*n* = 5876 children 2–18 years and *n* = 9953 adults 19–99 years). Americans’ nutrient intake from food categories in “What We Eat in America” and the 2015–2020 Dietary Guidelines for Americans was determined using the Food and Nutrient Database for Dietary Studies. Food cost and the cost of nutrients were obtained from Center for Nutrition Promotion and Policy food cost database 2001–2002 and 2003–2004 (adjusted for inflation).

**Results:**

The daily mean cost of food was $4.74 ± 0.06 for children and $6.43 ± 0.06 for adults. “Protein foods” and “mixed dishes” were the two most expensive food categories (43–45% of daily food costs), while “grains,” “fruits,” and “vegetables” combined accounted for ~ 18% of the daily cost, and “milk and dairy” accounted for 6–12% of total daily food costs in both adults and children. “Milk and dairy” were the least expensive dietary sources of calcium and vitamin D in the American diet, while “grains” were the least expensive sources of iron and magnesium, and “protein foods” were the least expensive sources of choline. “Fruits” and “vegetables” were the least expensive sources of potassium and vitamin C, respectively, and “snacks and sweets” were the least expensive sources of vitamin E.

**Conclusion:**

“Milk and dairy” were inexpensive sources of three of the four nutrients of public health concern (calcium, vitamin D, and potassium), while “grains” were the least expensive source of fiber. The results of this work reinforce the importance of consuming a variety of nutrient-rich foods for cost-effective, sustainable eating patterns.

## Background

Despite living in a high-income country, many Americans still do not meet their nutrient needs. The 2015–2020 Dietary Guidelines for Americans (2015–2020 DGA) identifies vitamin A, vitamin D, vitamin E, vitamin C, calcium, iron (for certain age/gender groups), magnesium, choline, potassium, and fiber as “shortfall nutrients” for Americans due to low consumption. Intakes of vitamin D, calcium, potassium, and fiber are low enough that the 2015–2020 DGA refers to them as “nutrients of public health concern” [1]. One barrier that prevents some Americans from consuming sufficient nutrients from healthy eating patterns is cost. Healthy diets tend to be more expensive [2–4]. Yet, to be sustainable or even feasible, healthy eating patterns must be affordable.

Sustainable nutrition, an important emerging area of research within nutrition science, needs to reflect social, cultural, and economic considerations in addition to environmental ones. The Food and Agricultural Organization (FAO) of the United Nations defines sustainable diets as “those diets with low environmental impacts that contribute to food and nutrition security and to healthy life for present and future generations. Sustainable diets are protective and respectful of biodiversity and ecosystems, culturally acceptable, accessible, economically fair and affordable, nutritionally adequate, safe, and healthy, while optimizing natural and human resources” [5]. Some aspects of this definition, notably the economic feasibility and affordability of sustainable diets, have not been a focus of the current literature. Few studies have incorporated economic considerations into evaluations of health and the environment when developing “sustainable” eating patterns [6–8]. One study from the United Kingdom that did consider dietary cost while modeling diets with lower greenhouse gas emissions (GHGE) found that it was possible to develop lower-GHGE diets at all income levels but that the amounts of certain foods consumed in the modeled diets would differ by income group [9].

Much of the current literature addresses sustainable nutrition narrowly by focusing on the potential impact of dietary changes on specific environmental impact markers like GHGE. While GHGE from specific foods is an important part of sustainability, it is only one metric within one of the domains of sustainability outlined in FAO’s definition. There are other factors, including land use, water use, and biodiversity loss, that influence the environmental impact of a food as well as other factors (impact on nutrition, health, productivity, livelihoods, gender equity, food security) that influence sustainability more broadly. Some of the current literature advocates major shifts in eating patterns, especially reducing consumption of animal-source foods, based on their potential to address environmental concerns [9–14] without considering the impacts of changing eating patterns on other facets of sustainability. Animal-source foods, like all foods, do have an environmental impact and contribute approximately 14.5% of global anthropogenic GHGE [15]. Globally, milk products contribute 2.7% to GHGE [16].

However, making recommendations that account for only or primarily the environmental impact of foods does not account for how these recommendations may affect both the nutrient content and cost of the diet [11, 17]. For instance, milk and dairy foods are nutrient-rich [18] and, in the U.S., are an important food source for several shortfall nutrients, including vitamin A, vitamin D, calcium, magnesium, and potassium, for Americans ages 2 and older [19–21]. Previous research indicates that their nutrient profile is difficult to replace with other foods [22]. Other animal-source foods are also important sources of nutrients. Beef and poultry are among the most important sources of dietary protein for American adults and children [20, 21], and animal-source foods (beef and milk) as well as yeast bread/rolls are the most nutrient-dense sources of energy for American adults [20]. Because of their nutrient-density, animal-source foods are included in all three healthy eating patterns in the 2015–2020 DGA (Healthy Vegetarian, Healthy U.S.-Style, and Healthy Mediterranean-Style) [23]. Some animal-source foods like milk and eggs are affordable as well as nutrient-dense [24].

Given the importance of identifying foods that are both affordable and nutrient-dense, this study focuses on the cost to the American consumer of getting shortfall nutrients from different food groups, especially dairy foods, to assess the economic implications of the dietary shifts recommended in some of the current “sustainable nutrition” literature [10–14, 17]. Our hypotheses were that it would cost the consumer more to achieve nutrient recommendations when dairy foods were eliminated and that dairy foods would be an economical choice for the nutrients they provide.

## Methods

### Database and study population

We used the National Health and Nutrition Examination Survey (NHANES), a nationally representative, cross-sectional survey conducted by the National Center for Health Statistics (NCHS) in noninstitutionalized, civilian US population using a complex, multistage, probability sampling design [25]. The 24-h dietary recall data from subjects 2+ years of age participating in NHANES 2011–2012 and 2013–2014 surveys were combined for all analyses. Subjects < 2 years of age, females who were pregnant and/or lactating (because of unique nutrient requirements), and those with incomplete or unreliable 24-h recall data as judged by the USDA Food Surveys Research Group staff, were excluded from the analyses. Separate analyses were conducted for age groups 2–18 years (*n* = 5876) and 19–99 years (*n* = 9953) in the gender-combined US population. All participants or proxies provided written informed consent and the Research Ethics Review Board at the NCHS approved the survey protocol [25].

### Estimates of dietary intake

Dietary intake data with reliable 24-h recall dietary interviews (day 1 data only) using United States Department of Agriculture’s (USDA) automated multiple-pass method were used [25]. The nutrient intake from food groups in What We Eat in America (WWEIA) food categories (Table 1) and from food categories created by combining foods indicated as sources of specific nutrients in the 2015–2020 DGA (Appendices 10–13) [1] was determined using the Food and Nutrient Database for Dietary Studies (FNDDS) 2011–2012 and 2013–2014 [26, 27], in conjunction with USDA National Nutrient Database for Standard Reference releases 26 and 27 [28] for NHANES 2011–2012 and 2013–2014 participants, respectively.

**Table 1:** WWEIA major food groups and their subcomponents^a^

MILK AND DAIRY	GRAINS
• Milk	• Cooked Grains
• Flavored Milk	• Breads, Rolls, Tortillas
• Dairy Drinks and Substitutes	• Quick Breads and Bread Products
• Cheese	• Ready-to-Eat Cereals
• Yogurt	• Cooked Cereals
PROTEIN FOODS	SNACKS AND SWEETS
• Meats	• Savory Snacks
• Poultry	• Crackers
• Seafood	• Snack/Meal Bars
• Eggs	• Sweet Bakery Products
• Cured Meats/Poultry	• Candy
• Plant-based Protein Foods	• Other Desserts
MIXED DISHES	FRUIT
• Mixed Dishes - Meat, Poultry, Seafood	• Fruits
• Mixed Dishes - Grain-based	VEGETABLES
• Mixed Dishes - Asian	• Vegetables, excluding Potatoes
• Mixed Dishes - Mexican	• White Potatoes
• Mixed Dishes - Pizza	BEVERAGES, NONALCOHOLIC
• Mixed Dishes - Sandwiches (single code)	• 100% Juice
• Mixed Dishes – Soups	• Diet Beverages
	• Sweetened Beverages
	• Coffee and Tea

^a^adapted from https://www.ars.usda.gov/ARSUserFiles/80400530/pdf/1314/Food_categories_2013-2014.pdf

A complete list of food codes in each food group is available at: https://www.ars.usda.gov/ARSUserFiles/80400530/apps/WWEIA1314_foodcat_FNDDS.xlsx

### Estimates of food cost

Food cost and the cost of nutrients were obtained from Center for Nutrition Promotion and Policy (CNPP) 2001–2002 and 2003–2004 cost databases [29, 30]. CNPP 2001–2002 and 2003–2004 databases provide cost per 100 g for food codes used in NHANES 2001–2002 and 2003–2004, respectively. NHANES food codes not in the cost database were hand-matched to a food code in the cost database. We matched food codes for NHANES 2001–2004 with NHANES 2011–2014 and linked them with CNPP 2001–2004 cost/100 g to obtain a base food cost. New food codes from the NHANES 2011–2014 that were not in the NHANES 2001–2004 dataset were hand-matched to the most similar available food codes.

Each NHANES food code was then mapped to one of 61 Consumer Price Index (CPI) food categories, and food costs for 2011–2014 were adjusted for inflation from 2003 to 2004 to 2011–2014 based on food category CPI over that time. The CPI associated with an NHANES cycle was obtained by averaging the monthly CPI values over the two-year period for the NHANES cycle. A small number of CPI series values were missing and were forecasted, back forecasted or interpolated using linear methods and values in the same series. The CPI food categories matched closely with the criteria for WWEIA food categories and FNDDS food categories, both of which group food codes. The mapping from NHANES food code to CPI food category then consisted of matching a CPI food category to a WWEIA or FNDDS category. The CPI provided cost adjustments for basic food categories, including alcoholic beverages but not mixed dishes. CPI information for mixed dishes was estimated by regressing CPI for non-mixed dish food codes on Food Products Equivalent Database (FPED) components by NHANES cycle and then using these regression coefficients and mixed dish FPED components to obtain CPI for mixed dishes. The updated food cost database was then used to determine nutrient cost for all food groups using the USDA food category system.

We then used the listing of top sources of calcium, potassium, dietary fiber, and vitamin D in Appendices 10–13, respectively, of the 2015–2020 DGA [1] and created a consumption frequency weighted composite of each food listed in these appendices and its associated cost (some groups were combined to reduce the number of foods to be listed, e.g., potatoes and sweet potatoes were combined as were several varieties of fish). For a few foods listed in these appendices (e.g., cod liver oil, raw mustard greens) there was no consumption in NHANES and, as such, these foods were not included.

### Statistics

All analyses were adjusted for the complex sample design of NHANES using appropriate survey weights, strata, and primary sampling units. Day one dietary weights were used in all intake analyses. All statistical analyses were performed with SAS software (version 9.2; SAS Institute Inc., Cary, NC) and SUDAAN (version 11; Research Triangle Institute; Raleigh, NC). SAS Proc SQL and SAS Macro programs were used for the general data manipulation and procedural coding. SUDAAN Proc Descript was used for all means analyses including mean nutrient amount from food group, percentage of consumers, and mean total daily cost from food group. SUDAAN Proc Ratio was used for all proportions analyses including mean nutrient cost per unit from food group, proportion daily total from food group for nutrients, and mean proportion total daily cost from food group. The underlying cost data was produced based on CNPP costs and CPI using SAS except for the use of SUDAAN Proc Reg to obtain the regression coefficients of CPI on FPED components.

## Results

The mean daily cost of food was $4.74 ± 0.06 for children and $6.43 ± 0.06 for adults. “Protein foods” and “mixed dishes” were the top two most expensive food groups, accounting for about 43–45% of daily food costs for both adults and children (totaling about $2.01 for children and $2.87 for adults; Table 2). “Grains,” “fruits,” and “vegetables” combined accounted for ~ 18% of the daily cost (totaling $0.86 for children and $1.15 for adults), and “snacks and sweets” accounted for about 9–14% of total daily cost ($0.67 for children and $0.57 for adults). “Milk and dairy” comprised 6–12% of total daily food costs, about $0.54 for children and $0.39 for adults (Table 2).

**Table 2:** Consumers and daily cost of major WWEIA food groups

Main Groups	% Consumers Mean ± SE	Total Cost Daily ($) Mean ± SE	% of Total Cost Mean ± SE
Age 2–18 years (*n* = 5876)			
All	100	4.74 ± 0.06	100
Milk and Dairy	82.0 ± 0.9	0.54 ± 0.01	11.5 ± 0.3
Protein Foods	75.1 ± 1.1	0.85 ± 0.03	18.0 ± 0.7
Mixed Dishes	77.5 ± 1.0	1.16 ± 0.03	24.6 ± 0.6
Grains	82.7 ± 0.9	0.30 ± 0.01	6.44 ± 0.15
Snacks and Sweets	87.8 ± 0.9	0.67 ± 0.02	14.2 ± 0.4
Fruit	51.1 ± 1.5	0.33 ± 0.01	7.05 ± 0.31
Vegetables	53.8 ± 1.3	0.23 ± 0.01	4.88 ± 0.17
Age 19–99 years (*n* = 9953)			
All	100	6.43 ± 0.06	100
Milk and Dairy	66.1 ± 0.8	0.39 ± 0.01	6.14 ± 0.13
Protein Foods	80.7 ± 0.6	1.46 ± 0.04	22.8 ± 0.6
Mixed Dishes	71.1 ± 0.8	1.41 ± 0.02	21.9 ± 0.4
Grains	78.7 ± 0.6	0.31 ± 0.01	4.83 ± 0.11
Snacks and Sweets	78.6 ± 0.6	0.57 ± 0.02	8.86 ± 0.26
Fruit	43.2 ± 1.0	0.33 ± 0.01	5.08 ± 0.14
Vegetables	68.0 ± 0.9	0.51 ± 0.02	7.88 ± 0.23

Data from NHANES 2011–2014

The daily intake and the unit cost of shortfall vitamins and minerals from different food groups are presented in Tables 3 and 4. The “milk and dairy” group was the main dietary contributor of calcium and vitamin D and also the least expensive dietary source of these nutrients for both children and adults. The “protein foods” and “grains” groups were the main dietary sources and the least expensive sources of choline and iron, respectively. The “grains” group was also the least expensive source of magnesium, while the “fruits” and “vegetables” groups were the least expensive sources of vitamin C and potassium, respectively. The “snacks and sweets” group was the least expensive source of vitamin E. Although the “milk and dairy” group was not the least expensive source of magnesium, potassium, and vitamin A, it was the second least expensive source and another cost-effective source of these nutrients.

**Table 3:** Percent daily intake and per unit cost of shortfall minerals by major WWEIA food groups

Main Groups	Calcium		Iron		Magnesium		Potassium	
	% Daily Intake Mean ± SE	Cost cents per mg Mean ± SE	% Daily Intake Mean ± SE	Cost cents per mg Mean ± SE	% Daily Intake Mean ± SE	Cost cents per mg Mean ± SE	% Daily Intake Mean ± SE	Cost cents per mg Mean ± SE
Age 2–18 years (*n* = 5876)								
All	100	0.45 ± 0.01	100	33.9 ± 0.5	100	2.01 ± 0.02	100	0.22 ± 0.002
Milk and Dairy	44.7 ± 0.7	0.12 ± 0.001	2.14 ± 0.08	182 ± 5	16.3 ± 0.4	1.42 ± 0.02	21.1 ± 0.5	0.12 ± 0.002
Protein Foods	3.33 ± 0.15	2.45 ± 0.10	10.1 ± 0.4	60.2 ± 1.6	12.8 ± 0.6	2.83 ± 0.11	14.0 ± 0.4	0.28 ± 0.01
Mixed Dishes	21.1 ± 0.7	0.53 ± 0.01	25.0 ± 0.6	33.3 ± 0.5	20.3 ± 0.6	2.43 ± 0.03	19.9 ± 0.5	0.27 ± 0.004
Grains	10.90 ± 0.2	0.27 ± 0.01	39.2 ± 0.8	5.56 ± 0.18	13.7 ± 0.3	0.95 ± 0.02	6.27 ± 0.16	0.22 ± 0.003
Snacks and Sweets	7.15 ± 0.25	0.90 ± 0.02	14.7 ± 0.5	32.8 ± 0.9	13.4 ± 0.4	2.13 ± 0.05	10.1 ± 0.4	0.30 ± 0.01
Fruit	0.96 ± 0.05	3.33 ± 0.05	1.51 ± 0.07	158 ± 3	4.15 ± 0.17	3.41 ± 0.07	7.00 ± 0.26	0.22 ± 0.004
Vegetables	1.96 ± 0.12	1.13 ± 0.05	2.76 ± 0.12	60.0 ± 1.4	5.58 ± 0.21	1.76 ± 0.04	9.05 ± 0.34	0.12 ± 0.003
Age 19–99 years (*n* = 9953)								
All	100	0.66 ± 0.01	100	42.5 ± 0.4	100	2.08 ± 0.01	100	0.24 ± 0.002
Milk and Dairy	30.9 ± 0.4	0.13 ± 0.001	1.23 ± 0.04	211 ± 6	6.87 ± 0.15	1.86 ± 0.03	8.78 ± 0.20	0.17 ± 0.003
Protein Foods	5.71 ± 0.12	2.62 ± 0.06	14.7 ± 0.3	65.6 ± 1.3	17.2 ± 0.3	2.76 ± 0.06	17.8 ± 0.3	0.30 ± 0.004
Mixed Dishes	21.9 ± 0.5	0.66 ± 0.01	24.8 ± 0.4	37.5 ± 0.3	17.4 ± 0.3	2.61 ± 0.02	19.3 ± 0.4	0.27 ± 0.003
Grains	11.2 ± 0.2	0.28 ± 0.01	33.1 ± 0.5	6.18 ± 0.08	13.4 ± 0.3	0.75 ± 0.01	6.02 ± 0.13	0.19 ± 0.002
Snacks and Sweets	7.25 ± 0.26	0.80 ± 0.02	11.5 ± 0.2	32.7 ± 0.7	9.93 ± 0.28	1.85 ± 0.02	7.94 ± 0.23	0.26 ± 0.004
Fruit	0.97 ± 0.03	3.45 ± 0.05	1.37 ± 0.04	158 ± 3	3.43 ± 0.08	3.08 ± 0.05	5.98 ± 0.15	0.20 ± 0.004
Vegetables	4.27 ± 0.16	1.21 ± 0.03	5.35 ± 0.17	62.5 ± 0.9	8.37 ± 0.22	1.96 ± 0.03	13.8 ± 0.3	0.13 ± 0.002

Data from NHANES 2011–2014

**Table 4:** Percent daily intake and per unit cost of shortfall vitamins by major WWEIA food groups

Main Groups	Choline		Vitamin A		Vitamin C		Vitamin D		Vitamin E	
	% Daily Intake Mean ± SE	Cost cents per mg Mean ± SE	% Daily Intake Mean ± SE	Cost cents per μg Mean ± SE	% Daily Intake Mean ± SE	Cos cents per mg Mean ± SE	% Daily Intake Mean ± SE	Cost cents per μg Mean ± SE	% Daily Intake Mean ± SE	Cost cents per mg Mean ± SE
Age 2–18 Years (*n* = 5876)										
All	100	2.18 ± 0.04	100	0.79 ± 0.01	100	6.16 ± 0.15	100	83.2 ± 1.6	100	67.2 ± 1.5
Milk and Dairy	14.0 ± 0.5	1.79 ± 0.03	31.3 ± 0.7	0.29 ± 0.004	0.99 ± 0.08	71.2 ± 5.2	66.0 ± 0.7	14.5 ± 0.2	3.08 ± 0.16	250 ± 11
Protein Foods	45.5 ± 1.0	0.86 ± 0.03	5.38 ± 0.36	2.66 ± 0.20	0.64 ± 0.06	173 ± 12	11.4 ± 0.7	131 ± 8	18.6 ± 1.0	65.1 ± 4.5
Mixed Dishes	27.4 ± 0.7	1.96 ± 0.05	17.4 ± 0.6	1.12 ± 0.03	7.16 ± 0.33	21.2 ± 0.87	6.28 ± 0.23	326 ± 15	23.7 ± 0.8	69.7 ± 1.1
Grains	3.03 ± 0.26	4.65 ± 0.34	20.1 ± 0.6	0.25 ± 0.01	4.92 ± 0.50	8.07 ± 0.82	10.5 ± 0.5	50.9 ± 2.5	10.4 ± 0.9	41.6 ± 4.2
Snacks and Sweets	7.15 ± 0.33	4.34 ± 0.13	7.33 ± 0.34	1.54 ± 0.06	5.53 ± 0.38	15.8 ± 0.78	1.34 ± 0.09	882 ± 59	23.8 ± 0.9	40.1 ± 1.3
Fruit	0.01 ± 0.005	1871 ± 1135	2.02 ± 0.13	2.77 ± 0.16	18.7 ± 1.0	2.32 ± 0.05	0.002 ± 0.001	99,900 ± 0	2.89 ± 0.19	164 ± 6
Vegetables	0.59 ± 0.06	18.0 ± 1.8	11.5 ± 0.8	0.34 ± 0.02	11.1 ± 0.8	2.72 ± 0.19	0.79 ± 0.08	512 ± 51	6.65 ± 0.26	49.3 ± 1.6
Age 19–99 Years (*n* = 9953)										
All	100	2.23 ± 0.03	100	0.98 ± 0.03	100	7.91 ± 0.13	100	136 ± 3	100	70.1 ± 0.7
Milk and Dairy	7.34 ± 0.14	1.86 ± 0.03	16.8 ± 0.5	0.36 ± 0.01	0.43 ± 0.03	113 ± 6	40.0 ± 0.7	20.9 ± 0.4	2.61 ± 0.20	165 ± 12
Protein Foods	53.8 ± 0.6	0.94 ± 0.02	9.96 ± 1.32	2.25 ± 0.37	1.12 ± 0.07	161 ± 9	29.7 ± 1.1	104 ± 5	23.4 ± 0.6	68.1 ± 2.0
Mixed Dishes	26.7 ± 0.5	1.82 ± 0.03	17.9 ± 0.5	1.20 ± 0.02	10.2 ± 0.4	17.0 ± 0.4	9.82 ± 0.46	303 ± 14	20.3 ± 0.4	75.6 ± 0.8
Grains	1.53 ± 0.11	7.01 ± 0.42	12.4 ± 0.5	0.38 ± 0.01	2.85 ± 0.13	13.4 ± 0.6	8.44 ± 0.32	77.8 ± 2.3	8.55 ± 0.31	39.6 ± 1.4
Snacks and Sweets	6.10 ± 0.23	3.23 ± 0.09	7.45 ± 0.35	1.17 ± 0.05	3.33 ± 0.24	21.0 ± 1.2	2.21 ± 0.15	545 ± 28	17.1 ± 0.5	36.2 ± 0.8
Fruit	0.01 ± 0.003	1181 ± 318	1.78 ± 0.12	2.80 ± 0.16	16.5 ± 0.5	2.43 ± 0.05	0.01 ± 0.002	99,900 ± 0	2.12 ± 0.07	168 ± 4
Vegetables	1.10 ± 0.08	16.0 ± 1.3	22.0 ± 1.2	0.35 ± 0.01	22.9 ± 0.9	2.72 ± 0.07	1.46 ± 0.11	735 ± 56	10.6 ± 0.4	52.0 ± 0.9

Data from NHANES 2011–2014

The 2015–2020 DGA [1] lists the primary food sources of calcium, potassium, vitamin D, and fiber, the four nutrients of public health concern, in appendices 10–13. Tables 5, 6, 7, and 8 present the estimated contribution of different foods to the percent daily intake of these nutrients as well as the per unit cost of these four nutrients from individual foods. Cheese and milk were the least expensive food sources of calcium. Orange juice, non-dairy milk, and “soy milk and tofu” were slightly more expensive sources of calcium. Fortified cereals, sardines, and yogurt were the most expensive sources. Per unit cost of calcium from these foods was more than 100 to 150% higher than from milk (Table 5). Milk was also the least expensive source of vitamin D. Eggs, fortified cereals, margarines, and soy milk were the next least expensive sources. The per unit cost of vitamin D from these foods was about 100% higher than vitamin D from milk (Table 6). “Potatoes and sweet potatoes,” milk, and juice were the main dietary and the least expensive sources of potassium (Table 7). The least costly sources of fiber were quinoa, chickpeas, and pearled barley followed by popcorn. The per unit cost of fiber from popcorn was over 100% higher than the fiber from the least expensive sources (Table 8).

**Table 5:** Percent intake and per unit cost of calcium in 2015–2020 DGA (appendix 11) identified food sources

Food Source	% Daily Intake Mean ± SE	Cost cents per mg Mean ± SE	Rank by Cost^a^	% Daily Intake Mean ± SE	Cost cents per mg Mean ± SE	Rank by Cost^a^
	Children 2–18 years			Adults 19–99 years		
Cheese	10.3 ± 0.4	0.11 ± 0.002	1	12.4 ± 0.5	0.12 ± 0.00	2
Fortified ready-to-eat cereals (various)	2.60 ± 0.16	0.36 ± 0.02	8	1.70 ± 0.10	0.49 ± 0.03	8
Milk (dairy)	31.0 ± 0.7	0.11 ± 0.001	1	14.4 ± 0.5	0.11 ± 0.00	1
Mustard Spinach (tender green), raw	NA	NA		NA	NA	
Orange Juice	2.48 ± 0.22	0.14 ± 0.004	3	2.19 ± 0.20	0.15 ± 0.00	3
Other non-dairy milk	1.10 ± 0.16	0.17 ± 0.01	4	1.39 ± 0.15	0.17 ± 0.00	4
Sardines, canned in oil, drained	0.001 ± 0.001	0.29 ± 0.003	7	0.01 ± 0.005	0.30 ± 0.00	7
Soy Milk and Tofu	0.32 ± 0.08	0.19 ± 0.004	5	0.48 ± 0.05	0.22 ± 0.01	5
Yogurt	2.02 ± 0.16	0.25 ± 0.003	6	2.21 ± 0.14	0.26 ± 0.00	6

Data from NHANES 2011–2014; NA – Intake data was not available in NHANES 2011–2014

^a^Ranking of foods from least expensive to most expensive source

**Table 6:** Percent intake and per unit cost of vitamin D in 2015–2020 DGA (appendix 12) identified food sources

Food Source	% Daily Intake Mean ± SE	Cost cents per μg Mean ± SE	Rank by Cost^a^	% Daily Intake Mean ± SE	Cost cents per μg Mean ± SE	Rank by Cost^a^
	Children 2–18 years			Adults 19–99 years		
Cod liver oil	NA	NA		NA	NA	
Eggs	4.61 ± 0.27	18.5 ± 0.3	2	8.80 ± 0.28	19.9 ± 0.3	2
Fish	3.18 ± 0.58	40.7 ± 4.4	7	15.5 ± 1.3	31.0 ± 2.4	6
Fortified ready-to-eat cereals (various)	8.98 ± 0.42	19.2 ± 0.6	3	6.57 ± 0.29	26.5 ± 1.0	5
Margarine	0.17 ± 0.04	21.0 ± 1.9	4	0.43 ± 0.04	22.5 ± 0.6	4
Milk (dairy)	57.7 ± 0.7	10.7 ± 0.11	1	30.2 ± 0.8	11.0 ± 0.2	1
Mushroom	NA	NA		NA	NA	
Orange Juice	1.86 ± 0.19	33.9 ± 1.3	6	1.78 ± 0.17	38.5 ± 1.3	7
Other non-dairy milk	0.79 ± 0.15	44.5 ± 6.6	8	1.20 ± 0.18	40.5 ± 3.5	8
Pork cooked	0.45 ± 0.07	190 ± 17	10	0.95 ± 0.09	158 ± 8	10
Soy Milk	0.46 ± 0.11	21.4 ± 0.2	5	0.79 ± 0.09	20.9 ± 0.1	3
Yogurt	1.38 ± 0.12	67.7 ± 2.9	9	1.51 ± 0.12	77.4 ± 4.1	9

Data from NHANES 2011–2014; NA – Intake data on cod liver oil and for irradiated mushroom were not available in NHANES 2011–2014

^a^Ranking of foods from least expensive to most expensive source

**Table 7:** Percent intake and per unit cost of potassium in 2015–2020 DGA (appendix 12) identified food sources

Food Source	% Daily Intake Mean ± SE	Cost cents per mg Mean ± SE	Rank by Cost^a^	% Daily Intake Mean ± SE	Cost cents per mg Mean ± SE	Rank by Cost^a^
	Children 2–18 years			Adults 19–99 years		
Avocado	0.12 ± 0.04	0.10 ± 0.0002	4	0.43 ± 0.04	0.10 ± 0.0001	5
Beans	1.33 ± 0.18	0.10 ± 0.01	4	1.98 ± 0.10	0.11 ± 0.01	6
Beet green	NA	NA		NA	NA	
Dried Peaches, prunes, apricots	0.004 ± 0.002	0.28 ± 0.14	8	0.03 ± 0.01	0.09 ± 0.001	3
Fish	0.62 ± 0.09	0.54 ± 0.02	9	1.65 ± 0.15	0.50 ± 0.01	9
Juice	5.68 ± 0.24	0.08 ± 0.002	2	3.65 ± 0.16	0.08 ± 0.01	2
Milk (dairy)	18.2 ± 0.4	0.09 ± 0.001	3	6.31 ± 0.20	0.09 ± 0.001	3
Potatoes and sweet potatoes	5.32 ± 0.27	0.06 ± 0.001	1	6.23 ± 0.19	0.07 ± 0.001	1
Spinach	0.13 ± 0.03	0.15 ± 0.003	6	0.42 ± 0.05	0.16 ± 0.01	7
Tomato paste, puree	NA	NA		NA	NA	
Yogurt	1.24 ± 0.10	0.20 ± 0.002	7	1.04 ± 0.06	0.19 ± 0.003	8

Data from NHANES 2011–2014; NA – Intake data was not available in NHANES 2011–2014

^a^Ranking of foods from least expensive to most expensive source

**Table 8:** Percent intake and per unit cost of fiber in 2015–2020 DGA (appendix 12) identified food sources

Food Source	% Daily Intake Mean ± SE	Cost cents per g Mean ± SE	Rank by Cost^a^	% Daily Intake Mean ± SE	Cost cents per g Mean ± SE	Rank by Cost^a^
	Children 2–18 years			Adults 19–99 years		
Artichoke	NA	NA		0.11 ± 0.03	13.0 ± 0.9	16
Avocado	0.25 ± 0.09	7.19 ± 0.02	7	0.93 ± 0.09	7.19 ± 0.01	7
Beans	3.63 ± 0.39	6.00 ± 0.51	5	5.56 ± 0.28	6.14 ± 0.50	5
Berries	1.31 ± 0.17	27.3 ± 0.4	21	1.09 ± 0.09	27.9 ± 0.19	22
Chickpeas	0.03 ± 0.01	1.81 ± 0.29	2	0.12 ± 0.03	1.61 ± 0.09	2
Collards	0.03 ± 0.01	21.9 ± 0.7	18	0.14 ± 0.03	20.4 ± 0.3	21
Dates	0.001 ± 0.001	10.4 ± 0	12	0.02 ± 0.01	10.4 ± 0.1	12
High fiber bran cereal	4.31 ± 0.28	9.90 ± 0.33	11	4.30 ± 0.17	7.84 ± 0.29	8
Mixed vegetables	0.41 ± 0.06	13.5 ± 1.2	16	1.06 ± 0.10	13.1 ± 0.9	17
Orange, banana, guava	3.67 ± 0.24	18.4 ± 0.7	17	3.42 ± 0.11	14.9 ± 0.4	18
Parsnips, winter squash	0.001 ± 0.001	24.5 ± 6.4	20	0.05 ± 0.02	15.6 ± 1.2	19
Pear, Apple	4.92 ± 0.27	12.4 ± 0.1	14	2.96 ± 0.16	11.8 ± 0.1	13
Pearled barley	0.01 ± 0.01	2.46 ± 0.01	3	0.03 ± 0.01	2.47 ± 0.01	3
Pecans, hazelnut, pistachio	0.03 ± 0.01	13.5 ± 0.8	15	0.25 ± 0.04	12.6 ± 0.5	15
Popcorn	1.72 ± 0.16	5.30 ± 0.19	4	1.13 ± 0.08	5.59 ± 0.20	4
Potatoes	4.57 ± 0.24	11.4 ± 0.1	13	4.94 ± 0.19	11.9 ± 0.1	14
Prunes, dried figs, pears	0.001 ± 0.0004	9.04 ± 0.22	9	0.05 ± 0.02	9.90 ± 0.36	11
Pumpkin	0.001 ± 0.001	23.5 ± 0.07	19	0.01 ± 0.001	17.6 ± 2.1	20
Pumpkin seeds, sunflower seeds	0.17 ± 0.06	6.78 ± 0.19	6	0.32 ± 0.05	8.86 ± 1.15	9
Quinoa	0.02 ± 0.01	1.58 ± 0.01	1	0.09 ± 0.03	1.53 ± 0.01	1
Wheat Cereal	1.87 ± 0.14	9.85 ± 0.37	10	0.92 ± 0.09	9.68 ± 0.60	10
Whole wheat bread	0.02 ± 0.01	9.01 ± 2.91	8	0.06 ± 0.02	6.23 ± 0.76	6

Data from NHANES 2011–2014; NA – Intake data was not available in NHANES 2011–2014

^a^Ranking of foods from least expensive to most expensive source

Adding a serving of the lowest-cost sources of the four nutrients of public health concern to the diet (i.e. one serving each of milk, potatoes and sweet potatoes, and quinoa) would increase the cost of the daily diet by approximately $0.81 for children and $0.88 for adults and would add about 350 cal. Hopefully, these additional calories could be traded off for other less nutrient dense foods. Additionally, several of these foods provide more than just the single public health nutrient. For example, while milk is the least expensive food source of calcium and vitamin D, it also provides potassium and additional nutrients, while quinoa, the least expensive source of dietary fiber, also provides some potassium.

## Discussion

Many Americans do not choose eating patterns that are consistent with dietary recommendations of 2015–2020 DGA [1]. Many factors including education, convenience, and accessibility affect eating patterns. Household income also affects food choice, nutrient intake, and nutrient adequacy, and observational studies from the U.S. indicate that nutritionally adequate diets tend to be more expensive than less-healthy diets [31, 32]. Americans in the lowest income quintiles spent three to six times more of their income on food (28.8–42.6% of annual before-tax income) than Americans in the highest income quintile (6.5–9.2%) over the last 25 years [33]. Americans with lower incomes are less likely to consume nutrient-adequate diets than Americans with higher incomes [32, 34, 35]. Even Americans who have relatively high incomes (up to 350% of the poverty income ratio) do not consume enough micronutrients, even though they can afford higher-quality diets than food insecure Americans [34]. Most American households, including food insecure households, spend more on food than what is allotted by the Thrifty Food Plan (TFP), the USDA-developed national standard for a minimal-cost diet that meets dietary recommendations [36]. On average, American households spend 24% more on food than the TFP allots [36]. The 2015–2020 DGA indicates that low intake of shortfall nutrients in the U.S. occurs because of unhealthy eating patterns, and it recommends adopting healthier eating patterns to bring intakes of shortfall nutrients closer to recommendations [1]. However, Americans need to be able to choose healthy eating patterns and meet their meet nutrient needs in a cost-effective manner. For healthy eating patterns to be implementable and sustainable long-term, they also need to be affordable.

This study indicates that foods from certain food groups, including the “milk and dairy,” “grains,” and “vegetables” groups were key dietary sources of nutrients of public health concern as well as the least expensive sources of these nutrients. Among individual foods from the 2015–2020 DGA, milk and cheese were the least expensive sources of calcium, and milk was the least expensive source of vitamin D. Quinoa was the lowest cost source of fiber among foods from the 2015–2020 DGA, followed by chickpeas and pearled barley. Several food sources of potassium were relatively low-cost, including potatoes and sweet potatoes, juice, and milk (all under 0.10 cents/mg). Increasing intake of milk and dairy foods to meet recommendations from the 2015–2020 DGA could increase intake of several important nutrients in the diet in an inexpensive manner. Results of a recent dietary modeling analysis support this assertion and show that increasing dairy intake increases calcium, magnesium, potassium, vitamin A, and vitamin D intake and decreases the prevalence of inadequacy of these nutrients [37]. Similarly, increasing intake of quinoa, chickpeas, and potatoes and sweet potatoes could help increase intake of potassium and fiber at a low cost to the consumer.

Fortification plays an important role in providing low-cost nutrients [38]. For instance, vitamin A is added to lower fat milks to replace the amount found in whole milk and vitamin D is added to all milk, which helps it serve as an affordable source of these nutrients in the U.S. diet. Similarly, refined grains and several ready-to-eat cereals are fortified with iron as well as other minerals and B vitamins. Fortification is responsible for the “grains” food group being the lowest cost source of iron for both children and adults and for fortified ready-to-eat cereals being an inexpensive source of vitamin D. Since refined grains and ready-to-eat cereals do not naturally contain these nutrients, fortification helps provide an accessible, affordable source for these shortfall nutrients.

This study has several strengths and limitations. Using a large, nationally representative database to estimate nutrient cost is a major strength of the present study. Comparing nutrient cost by both WWEIA food categories and food sources specified by 2015–2020 DGA is an additional strength. However, using self-reported 24-h dietary recall data may be a limitation, as it is subject to over- or under-reporting. Another limitation of the study was the use of a food price database that may not reflect geographic or location-related differences in food price and diet cost. Additionally, some food codes in NHANES 2011–2014 had to be hand matched to the closest available food codes in NHANES 2001–2004 database, which was linked to the CNPP food cost database. This process may have introduced inaccuracies in cost estimations and as such updated cost information relevant for NHANES should be collected.

## Conclusions

Dietary cost is an important yet often overlooked aspect of sustainable eating patterns. “Milk and dairy” were the least expensive dietary sources of calcium and vitamin D, while “grains” were the least expensive sources of iron and magnesium, and “protein foods” were the least expensive sources of choline. “Fruits” and “vegetables” were the least expensive sources of potassium and vitamin C, respectively, while “snacks and sweets” were the least expensive sources of vitamin E. The results of this work reinforce the importance of consuming a variety of nutrient-rich foods for cost-effective and sustainable eating patterns.

## Data Availability

The datasets analyzed during the current study are available in the Center for Disease Control and Prevention repository; National Health and Nutrition Examination Survey (2011–2012 and 2013–2014).

## Abbreviations

2015–2020 DGA: 2015–2020 Dietary Guidelines for Americans
CNPP: Center for Nutrition Promotion and Policy
CPI: Consumer Price Index
FAO: Food and Agricultural Organization
FNDDS: Food and Nutrient Database for Dietary Studies
FPED: Food Products Equivalent Database
GHGE: greenhouse gas emissions
NCHS: National Center for Health Statistics
NHANES: National Health and Nutrition Examination Survey
USDA: United States Department of Agriculture
WWEIA: What We Eat in America
